# Characterizing Mild Behavioral Impairment and Its Relationship to Cognition in Community‐dwelling older Latinos from the Boston Latino Aging Study

**DOI:** 10.1002/alz.086966

**Published:** 2025-01-03

**Authors:** Jorge Alcina, Diana Munera, Alex L Badillo‐Cabrera, Nikole A Bonillas Félix, Lusiana Martinez, Averi Giudicessi, Elizabeth Kaplan, Jairo E. Martinez, Clara Vila‐Castelar, Nadine A Schwab, Liliana A Ramirez Gomez, Marta Gonzalez Catalan, Daniel G Saldana, Jennifer R. Gatchel, Yakeel T. Quiroz

**Affiliations:** ^1^ Massachusetts General Hospital, Harvard Medical School, Boston, MA USA; ^2^ McLean Hospital, Belmont, MA USA

## Abstract

**Background:**

Latinos represent the fastest‐growing subpopulation of U.S. older adults and are 1.5 times more likely than non‐Latino Whites to be diagnosed with Alzheimer’s disease and related dementias. Mild Behavioral Impairment (MBI) describes emergence of new neuropsychiatric symptoms in individuals over 50, and is associated with late‐life depression, cognitive decline, and dementia. Despite its clinical relevance, the relationship between MBI and dementia risk is poorly understood in diverse populations. Here, we assessed MBI symptoms in older, community‐dwelling Latinos and examined the relationship of these symptoms to objective and subjective cognition.

**Method:**

53 participants from the Boston Latino Aging Study were included, ages 55 years and older, 71% female, mean age 64.13 ± 6.79 years, mean education 12.30 ± 5.12 years, 49 cognitively unimpaired, and 4 with mild cognitive impairment. Objective cognition was measured using the Preclinical Alzheimer’s Cognitive Composite‐5 (PACC5). Neuropsychiatric symptoms were measured with the Mild Behavioral Impairment Checklist (MBI‐C; self‐report) domains of interest motivation and drive (IMD) and mood anxiety (MA). Subjective cognitive decline was assessed with the Cognitive Function Instrument (CFI; self‐report). Associations among MBI‐C domains, CFI, and cognition were examined using Spearman’s correlation, controlling for age, sex, and education.

**Result:**

Mean total MBI‐C IMD domain score was 2.37 ± 3.59, mean total MBI‐C MA domain score was 2.45 ± 3.79 and mean total CFI score was 3.60 ± 3.51. Higher scores on the MBI‐C IMD were associated with higher CFI (r = .606, p < .001) and lower PACC scores (r = ‐.333, p = .015). Higher scores on the MBI‐C MA were associated with higher CFI (r = .384, p = .005). There were no other significant associations.

**Conclusion:**

Community dwelling older Latinos endorsed MBI symptoms of IMD and MA captured using the MBI‐checklist self‐report. MBI IMD symptoms were associated with greater subjective decline and worse objective cognition. Findings underscore the need for screening and management of neuropsychiatric symptomatology in Latino/a/e/x adults, and that such symptoms can be captured using the MBI‐checklist. Future longitudinal studies with larger samples are needed to further understand cognitive effects of neuropsychiatric functioning in Latino/a/e/x and other minoritized groups.

